# Supplementary material for: DNA Methylation as a Molecular Mechanism of Carcinogenesis in World Trade Center Dust Exposure: Insights from a Structured Literature Review
Source: Biomolecules. 2024 Oct 15;14(10):1302. doi: 10.3390/biom14101302 (PMC11506790; doi:10.3390/biom14101302)
Supplement: Supplementary file 1 [file biomolecules-14-01302-s001.zip › biomolecules-3221694-supplementary.pdf]

Supplement

Supplementary Table S1: Search strategy in PubMed

**Terms for WTC Carcinogens (MeSH)**	**AND**	**DNA Methylation (MeSH)**<br>**human**<br>**English[Language]**	**NOT**	**Prenatal OR Maternal**
Metals<br>Asbestos<br>Polychlorinated biphenyls<br>Polycyclic aromatic hydrocarbons<br>Dioxins<br>Benzene				

Supplementary Table S2: Literature on WTC dust components and DNA methylation in human populations (n=80 studies)

Author, Year (ref)	Study Design			Study Results*		
	**WTC Dust Carcinogen (Exposure)**	**Study design and participants, assessment of exposure to carcinogen(s)**	**Sample type, Methylation method, statistical analysis**	**Global Pattern of DNA Methylation**	**Cancer-related, Site-specific DNA methylation of *Oncogenes***	**Cancer-related, Site-specific DNA methylation of *Tumor Suppressors***
**HEAVY METALS**						
Takahashi, 2005[87]	Chromium (Cr)	8 lung tumors from chromate workers	FFPE tumors<br><br>DNA methylation-specific PCR of *MLH1 p*romoter	NA	NA	5/8 chromate lung tumors had DNA methylation of *MLH1* promoter
Chanda, 2006[56]	Arsenic (As)	Patients from arsenic clinic, Kolkata, India. Phase 1: *p53* in 96 samples, 24 of which were controls from the same areas Phase 2: *p16* in 62 samples, 11 of which were controls. Survey on prior As exposure from drinking water; water samples tested for As by atomic absorption spectrophotometer with hydride generation system.	Blood<br><br>DNA methylation-specific PCR of *p53* & *p16* promoters<br><br>Two-tail median test	NA	NA	Hypermethylation of p16^{INK4A} *(CDKN2A), TP53*
Kondo, 2006[86]	Chromium (Cr)	30 lung tumors from chromate workers; 38 non-chromate worker lung tumor controls.	FFPE tumors<br><br>DNA methylation-specific PCR of the *p16* promoter<br><br>χ^2 test	NA	NA	Hypermethylation of p16^{INK4A} *(CDKN2A)*
Chen, 2007[57]	Arsenic (As)	38 urothelial carcinomas; 17 of which from As contaminated areas (Taiwan southwestern townships).<br>As assessment NR.	FFPE tumors<br><br>DNA methylation-specific PCR of *DAPK* promoter<br><br>Mantel–Haenszel	NA	NA	Hypermethylation of *DAPK*
Pilsner, 2007[62]	Arsenic (As)	294 adults in Araihazar, Bangladesh with varying	blood	As exposure associated with	NA	NA

		exposure to As contaminated drinking water. Well-water and urinary As concentrations measured by graphite furnace atomic absorption.	DNA methyl acceptance capacity method<br><br>ANOVA	increased global DNA methylation		
Zhang, 2007[66]	Arsenic (As)	103 Chinese arsenicosis patients from coal containing high arsenic; 110 healthy controls.	blood<br><br>DNA methylation-specific PCR of the *P16* promoter<br><br>χ^2 test	NA	NA	Hypermethylation of p16^{INK4A} *(CDKN2A)*
Majumdar, 2010[60]	Arsenic (As)	64 Indian residents with arsenicosis and history of exposure to arsenic contaminated water plus clinical symptoms.	blood<br><br>DNA methyl acceptance capacity method<br><br>ANOVA	As exposure associated with increased global DNA methylation	NA	NA
Ali, 2011[84]	Chromium (Cr)	36 lung tumors from chromate workers; 25 non-chromate worker lung tumor controls.	FFPE tumors<br><br>DNA methylation-specific PCR of *APC* at 7 CpG sites, *hMLH1* at 7 CpG sites, *MGMT* at 7 CpG sites<br><br>ANOVA	NA	NA	Hypermethylation of *MGMT, APC*
Hou, 2011[72]	Arsenic (As) & Nickel (Ni)	63 Italian male workers (electric steel plant). Workplace metal airborne PM_{10} inductively coupled-plasma mass spectrometry.	blood<br><br>DNA methylation-specific PCR of *APC, p16, p53 & RASSF1A* promoters<br><br>Mixed model liner regression	NA	NA	As/Ni: Hypermethylation of p16^{INK4A} *(CDKN2A), APC*<br><br>Hypomethylation of *TP53, RASSF1A*
Smeester, 2011[64]	Arsenic (As)	6 individuals with elevated levels of As and signs of arsenicosis, Zimapan, Hidalgo, Mexico; 6 controls. As measured in urine, method NR.	blood<br><br>DNA Affymetrix Human Promoter 1.0R array	182/183 differentially methylated genes, hypermethylated in those with arsenicosis	NA	Hypermethylation of *PRDM2, SUFU*

Hossain, 2012[68]	Arsenic (As) & Cadmium (Cd)	202 females from Northern Argentina with varied As exposure, measured in urine using high performance liquid chromatography.	blood<br><br>DNA methylation-specific PCR of *p16* at 7 CpG sites, *MLH1* at 4 CpG sites, and *LINE1* at 4 CpG sites<br><br>Spearman's rank correlation	NA	Cd: Hypermethylation of *DNMT1*	As: Hypermethylation of *MLH1,* p16^{INK4A} *(CDKN2A)*<br><br>Cd: Hypermethylation of *MLH1*
Wang, 2012[88]	Chromium (Cr)	115 Chinese workers (chromate production plant). Red blood cell Cr concentration measured by inductively coupled plasma mass spectrometry.	blood<br><br>MethylFlash Methylated DNA Quantification Kit<br><br>Pearson or Spearman correlation	Cr exposure associated with decreased global DNA methylation	NA	NA
Bailey, 2013[55]	Arsenic (As)	16 female residents of Zimapán, Hildago, Mexico with varying exposure to As in drinking water. Urinary As measured by hydride generation atomic absorption spectrometry with cryotrapping.	blood<br><br>Affymetrix Human Promoter 1.0R array<br><br>Pearson correlation	As exposure associated with increased global DNA promoter methylation	NA	NA
Niedzwieck, 2013[61]	Arsenic (As)	379 residents of Araihazar, Bangladesh with varying exposure of As contaminated well water, measured in urine using high performance liquid chromatography.	blood<br><br>DNA [3H]-methyl incorporation assay<br><br>Spearman's correlation	As exposure associated with increased DNA global methylation	NA	NA
Tajuddin, 2013[74]	Arsenic (As) & Nickel (Ni)	892 participants from the Spanish Bladder Cancer/EPICURO study. Metal concentrations in toenails by inductively coupled plasma–mass spectrometry.	blood<br><br>DNA methylation-specific PCR of *LINE-1 at 4 CpG sites*<br><br>bivariate robust linear regression	As associated with decreased global DNA methylation<br><br>Ni associated with increased global DNA methylation	NA	NA
Fan, 2014[89]	Chromium (Cr) & Nickel (Ni)	66 male workers (boilermakers). Real-time $PM_{2.5}$ personal particle exposure	blood	Metal exposure associated with increased global	NA	NA

		continuously monitored using the light-scattering technology.	DNA methylation pyrosequencing in *Alu & LINE-1*<br><br>mixed-effects linear regression	DNA methylation		
Lu, 2014[59]	Arsenic (As)	40 arsenicosis cases from villages in Bameng, Inner Mongolia, China with high As exposure from contaminated drinking water, matched to 1) 40 controls with high As exposure but no arsenicosis and 2) 40 controls with minimal As exposure. No As measure	blood<br><br>DNA methylation-specific PCR of *p16*<br><br>logistic regression	NA	NA	Hypermethylation of p*16*^{INK4A} *(CDKN2A)*
Tellez-Plaza, 2014[67]	Arsenic (As)	24 participants with moderate As exposure, 24 with low As exposure; 16 from Arizona, 16 from Oklahoma, and 16 from North or South Dakota, as part of the Strong Heart Study.	blood<br><br>MethylFlash Methylated DNA Quantification Kit<br><br>linear regression	As exposure associated with decreased global DNA methylation	NA	NA
Wong, 2014[71]	Arsenic (As) & Cadmium (Cd)	87 males from the Harvard Boilermakers Longitudinal Study. Metals measured in toenails by dynamic reaction cell-inductively coupled plasma mass spectrometry.	blood<br><br>DNA methylation pyrosequencing in *Alu & LINE-1*<br><br>Linear-mixed effects regression	No association between exposure from welding fumes and global DNA methylation	NA	NA
Yang, 2014[65]	Arsenic (As)	14 urothelial carcinoma patients living in arsenicosis-endemic areas of southwestern Taiwan. 14 unexposed urothelial carcinoma patients. As exposure: concentration of As in available drinking water x years drinking the water.	frozen urothelial carcinoma tissues<br><br>Infinium Human Methylation27 BeadChip<br><br>Wilcoxon signed-rank test	208/231 sites had higher mean methylation levels among As exposed	Hypermethylation of *CTNNA*2	NA
Yang, 2014[90]	Nickel (Ni)	165 Chinese male workers (iron and steel); 67 unexposed controls. Urinary nickel measured by ammonium pyrrolidine dithiocarbamate extraction and atomic	blood<br><br>DNA methylation-specific PCR of *p15* & *p16*<br><br>logistic regression	NA	Hypermethylation of *p15 (ABL1)*	NA

		absorption with graphite furnace.				
Argos, 2015[54]	Arsenic (As)	413 residents of rural communities in central Bangladesh, from the Bangladesh Vitamin E and Selenium Trial, with varying As exposure as by total urinary arsenic concentration measured from graphite furnace atomic absorption spectrometry.	blood<br><br>Illumina Human Methylation 450K BeadChip<br><br>linear regression	No significant global DNA methylation patterns observed	NA	NA
Rager, 2015[63]	Arsenic (As)	46 female residents of Chihuahua, Mexico. Arsenic measured in urine using hydride generation-cryotrapping-atomic absorption spectrometry.	blood<br><br>Affymetrix Human Promoter 1.0R arrays<br><br>linear regression	NA	NA	Hypermethylation of *PRDM2*
Ameer, 2017[53]	Arsenic (As)	93 females from Northern Argentina with varying As exposure, measured as metabolites in urine using high performance liquid chromatography.	blood<br><br>Illumina Human Methylation 450K BeadChip<br><br>Spearman's rank correlation	Among the top 1000 CpGs, 87% had increased methylation	NA	Hypermethylation of *CREBBP*
Chung, 2017[77]	Cadmium (Cd)	209 urothelial carcinomas; 417 healthy controls. Metals measured in both blood and urine by inductively coupled plasma-mass spectrometry.	blood<br><br>Global 5-MedC levels detected by high-performance liquid chromatography<br><br>χ^2 test	Cd exposure associated with decreased levels DNA methylation in urine but not blood	NA	NA
Hossain, 2017[58]	Arsenic (As)	175 participants from arsenic-endemic areas of rural Bagledash; 61 controls from a non-endemic area. As measured in hair and toenails using inductively coupled plasma mass spectrometry.	blood<br><br>DNA methylation pyrosequencing in *LINE-1*<br><br>Kruskal-Wallis test and linear regression	As exposure associated with decreased global DNA methylation; more pronounced in females than males	NA	NA

Hu, 2018[85]	Chromium (Cr)	117 Chinese workers (chromate production plant); 30 unexposed controls. Cr measured in blood by inductively coupled plasma mass spectrometry	blood<br><br>DNA methylation-specific PCR of *MGMT, HOGG1, XRCC1, ERCC3 & RAD51*<br><br>linear regression	NA	NA	Hypermethylation of *MGMT*
Riffo-Campos, 2018[69]	Arsenic (As) & Cadmium (Cd)	73 participants of the Aragon Workers Health Study longitudinal cohort. Urinary metals measured by inductively coupled plasma-mass spectrometry.	blood<br><br>Illumina Human Methylation 450K BeadChip<br><br>regression matric	NR	As: *GNAS*	Cd: *ZBTB16, SMAD3, IGF2BP2, LARP4B, ARID1B, ARHGEF10*
Yang, 2019[76]	Beryllium (Be)	8 patients with chronic beryllium disease; 8 control beryllium-sensitized patients. Leftover lung cells collected from either a clinical or research bronchoscopy.	blood<br><br>Illumina Human Methylation 450K BeadChip<br><br>mixed-effects linear regression	All 1,334 significant CpGs are enriched for hypomethylatio n	Hypermethylation of *CXCR4*	NA
Domingo-Relloso, 2020[78]	Cadmium (Cd)	2,325 participants of the Strong Heart Study prospective cohort of American Indian adults. Cd measured in urine by inductively coupled plasma–mass spectrometry.	blood<br><br>Illumina MethylationEPIC BeadChip (850K)<br><br>linear regression	NR	Hypermethylation of *RARA*	NA
Lee, 2020[82]	Cadmium (Cd)	50 smokers and 50 non-smokers from the Korean National Health and Nutrition Examination Survey. Cd measured in the blood by graphite furnace atomic absorption spectrometry.	blood<br><br>Illumina Human Methylation 450K BeadChip<br><br>linear regression	NR	*RARA, NFE2L2, RET*	*GATA3, RET, CD79B, ETV6, TGFBR2, CBFB BRCA2*
Li, 2020[73]	Arsenic (As) & Nickel (Ni)	23 workers (e-waste); 23 residents living near e-waste sites; 45 unexposed controls. Metals measured in blood by inductively coupled plasma-mass spectrometry.	blood<br><br>DNA global 5-mc levels<br><br>linear regression	Neither As or Ni associated with global DNA methylation	NA	NA

Lin, 2020[83]	Cadmium (Cd)	531 participants from the YOung TAiwanese Cohort Study. Cd measured in urine by inductively coupled plasma-mass spectrometry.	blood<br><br>DNA global 5-methyl-2'-deoxycytidine expressed as a percentage of total cytosine content (5-mdC/dG)<br><br>linear regression	Cd exposure not associated with global DNA methylation.	NA	NA
Hasani Nourian, 2021[70]	Arsenic (As) & Cadmium (Cd)	69 relapsing–remitting multiple sclerosis patients; 69 healthy controls. Metals measured in blood by GBC atomic absorption spectrometer with graphite furnace.	blood<br><br>DNA methylation-specific PCR of *ACKR3 & APOE*<br><br>χ^2 test	NA	As/Cd: Hypomethylation of *ACKR3*	NA
Hsu, 2022[75]	Arsenic (As), Cadmium (Cd) & Chromium (Cr)	355 urothelial carcinomas from Taiwan; 710 healthy controls. Metal concentration measured in urine by inductively coupled plasma-mass spectrometry.	blood<br><br>DNA 5-methyl-2'-deoxycytidine (%5-MedC), as a proxy for DNA methylation<br><br>logistic regression	Joint effects of multiple metals associated with increased global DNA methylation	NA	NA
Issah, 2022[81]	Cadmium (Cd)	100 workers from Ghana (e-waste); 50 unexposed controls. Metals measured in whole blood by inductively coupled plasma mass spectrometry.	blood<br><br>DNA pyrosequencing in *LINE-1*<br><br>linear regression	Cd exposure associated with decreased global DNA methylation	NA	NA
Hsueh, 2023[79]	Cadmium (Cd)	218 chronic kidney disease patients; 422 controls. Metals measured in blood by inductively coupled plasma mass spectrometry.	blood<br><br>DNA 5-methyl-2'-deoxycytidine (5mdC) expressed as a percentage of the total cytosine content (methylated and non-methylated) as a proxy for DNA methylation<br><br>logistic regression	Cd exposure associated with increased global DNA methylation	NA	NA
Huang, 2023[80]	Cadmium (Cd)	174 renal cell carcinomas; 673 healthy controls. Cadmium measured in blood by	blood<br><br>DNA 5-methyl-2'-deoxycytidine (5mdC) expressed as a	Cd exposure associated with decreased	NA	NA

		inductively coupled plasma mass spectrometry.	percentage of the total cytosine content (methylated and non-methylated) as a proxy for DNA methylation<br><br>logistic regression	global DNA methylation		
**ASBESTOS**						
Andujar, 2010[91]	Asbestos	75 non-small-cell lung cancers. Asbestos exposure assessed by questionnaire on job history, including past occupational, domestic, and environmental exposure and by asbestos body counts in dry lung tissue.	FFPE lung tissue<br><br>DNA *M*ethylation-specific PCR of *RASSF1A, p16INK4a, RARβ, MGMT & DAPK*<br><br>logistic regression	NA	NA	Hypomethylation p*16*^{INK4A} *(CDKN2A)*
Fujii, 2012[92]	Asbestos	39 malignant pleural mesotheliomas, 46 lung cancers, 25 benign asbestos pleurisy and 30 other causes. Asbestos exposure assessed by occupational history with an in-person questionnaire or interview.	pleural fluid<br><br>Illumina Human Methylation 450K BeadChip<br><br>generalized linear regression	NA	NA	Hypermethylation p*16*^{INK4A} *(CDKN2A)*
Kettunen, 2017[93]	Asbestos	14 Caucasian male lung cancers exposed to asbestos; 14 unexposed lung cancers; 6 non-lung cancer controls. Asbestos exposure assessed by work histories interviews and pulmonary fiber counts by scanning electron microscopy with energy dispersive spectrometry.	Hematoxylin-Eosin-stained lung tissue<br><br>DNA *M*ethylation-specific PCR of *RASSF1A, p16INK4a, RARβ, MGMT & DAPK*<br><br>logistic regression	NA	NA	Hypomethylation of *DFNA5 (GSDME), EDAR*
Yu, 2017[94]	Asbestos	47 healthy workers (plants that formerly manufactured asbestos-related products), 52 participants with benign asbestos-related disorders; 26 healthy unexposed controls. Asbestos exposure by occupational history records	frozen tissue<br><br>MethylFlash Methylated DNA Quantification Kit<br><br>linear regression	Asbestos exposure associated with decreased global DNA methylation	NA	NA

		and/or asbestos-related abnormal radiological findings.				
**POLYCYCLIC AROMATIC HYDROCARBONS**						
Zhang, 2006[124]	benzo[a]pyrene	40 hepatocellular carcinomas. PAH-DNA adducts in paraffin sections by monoclonal antibody 5D11 against benzo[a]pyrene diol epoxide (BPDE) modified DNA.	frozen tissue<br><br>DNA *M*ethylation-specific PCR of *p16*<br><br>Student's t and χ^2	NA	NA	Hypermethylation of p*16*^{INK4A} *(CDKN2A)*
Chao, 2008[118]	1-OHP, 8-oxodG	37 Taiwan male workers (coke oven), 15 high-exposure and 22 low-exposure. PAHs quantified in urine by liquid chromatography with tandem mass spectrometry.	urine<br><br>DNA methylation N7-MeG levels<br><br>Student's t and χ^2	PAH exposure associated with increased global DNA methylation	NA	NA
Pavanello, 2009[119]	1-pyrenol & anti-B[a]PDE	49 Polish male workers (coke oven); 43 unexposed male controls. PAHs quantified in urine by high-performance liquid chromatography–fluorescence.	blood<br><br>DNA pyrosequencing in *Alu & LINE-1. M*ethylation-specific PCR of *p53, HIC1& IL-6*<br><br>univariate linear regression	PAH exposure associated with increased global DNA methylation	NA	Hypomethylation of *TP53*
Pavanello, 2010[120]	1-pyrenol	48 Polish male workers (coke oven); 44 unexposed male controls. 1-Pyrenol quantified in urine by high-performance liquid chromatography–fluorescence.	blood<br><br>EZ-96 DNA Methylation-Gold Kit<br><br>univariate linear regression	NA	NA	Hypomethylation of *TP53*
Yang, 2012[116]	1-OHP	69 Chinese male workers (coke oven); 47 unexposed male controls. 1-OHP quantified in urine.	blood<br><br>DNA methylation-specific PCR of *p16*<br><br>Mann–Whitney *U* test	NA	NA	Hypermethylation of p*16*^{INK4A} *(CDKN2A)*
Alegría-Torres, 2013[114]	1-OHP	39 male workers (brickmakers). 1-OHP quantified in urine by high-performance liquid chromatography.	blood<br><br>DNA methylation-specific PCR of *p53, TNF-α, IFN-γ, IL-6,* and *IL-12*	NA	NA	Hypomethylation of p*16*^{INK4A} *(CDKN2A)*

			Mann–Whitney *U* test			
Duan, 2013[115]	1-OHP	82 Chinese workers (coke plant); 62 unexposed male controls. 1-OHP quantified in urine.	blood<br><br>DNA methylation-specific PCR of *LINE-1* and *MGMT* promoters<br><br>Mann–Whitney and Kruskal–Wallis	NA	NA	Hypomethylation of *MGMT*
White, 2015[111]	Polycyclic aromatic hydrocarbons	873 breast cancer cases from the Long Island Breast Cancer Study Project; 941 cancer-free, female controls. PAH-DNA adduct assays to quantify exposure.	blood<br><br>DNA methylation-specific PCR of 13 breast cancer-related genes *(APC, BRCA1, CCND2, CDH1, DAPK1, ESR1, GSTP1, HIN1, CDKN2A, PGR, RARβ, RASSF1A, and TWIST1)* promoters<br><br>logistic regression	PAH exposure associated with increased global methylation	NA	Hypermethylation of *APC*
Zhang, 2015[123]	B[a]P	74 Han male workers (coke oven); 47 unexposed male controls. B[a]P quantified in urine by high performance liquid chromatography. Occupational air samples also collected.	blood<br><br>DNA methylation-specific PCR of *p14(ARK), p15(INK4b) & p16(INK4a)*<br><br>Statistical analysis: χ^2 test	NA	NA	Hypermethylation of p*16*^{INK4A} *(CDKN2A)*
White, 2016[112]	Polycyclic aromatic hydrocarbons	1508 breast cancers from the Long Island Breast Cancer Study Project; 1556 cancer-free, female controls. PAH exposure from current smoking, residential ETS, grilled/smoked meat intake, and synthetic log burning assessed by questionnaire.	blood<br><br>DNA methylation-specific PCR of 13 breast cancer-related genes *(APC, BRCA1, CCND2, CDH1, DAPK1, ESR1, GSTP1, HIN1, CDKN2A, PGR, RARβ, RASSF1A, and TWIST1)* promoters. *LINE-1* and *LUMA* assays.<br><br>logistic regression	PAH exposure not associated with global methylation	Hypomethylation of *ESR1, CCND2*	Hypermethylation of *CDH1*<br><br>Hypomethylation of *BRCA1, DAPK*
Zhang, 2016[113]	1-OHNa, 2-OHNa,	117 male workers (diesel engine exhaust); 112 unexposed	blood	No association between PAH	NA	Hypomethylation of

	2-OHFlu,<br>2-OHPhe,<br>9-OHPhe,<br>1-OHP	controls. PAHs measured in urine by liquid chromatography with tandem mass spectrometry.	DNA methylation-specific PCR of *p16, RASSF1A, MGMT* promoters. Pyrosequencing in *LINE-1*<br><br>t-*test* and Mann-Whitney	exposure and global DNA methylation		p*16*^{INK4A} *(CDKN2A), MGMT*
Yang, 2018[121]	2-hydroxynaphthalene, 2-hydroxyfluorene, 9-hydroxyphenanthrene, 1-hydroxypyrene	348 workers (coke oven plant); 131 unexposed controls. Urinary PAH measured by high performance liquid chromatography.	blood<br><br>DNA Pyrosequencing in *LINE-1*<br><br>logistic regression	PAH exposure associated with decreased global DNA methylation	NA	NA
Silva, 2019[110]	Polycyclic aromatic hydrocarbons	59 Brazilian workers (construction); 49 unexposed controls. Work environment concentration of PAHs determined by gas chromatography/mass spectrometry.	blood<br><br>DNA methylation-specific PCR of *CDKN2A, MLH1, APC &* *LINE-1* promoters<br><br>t-*test* and Mann-Whitney	PAH exposure associated with decreased global methylation	NA	Hypermethylation of *MLH1, APC, p16*^{INK4A} *(CDKN2A)*
Meng, 2021[122]	B[a]P	109 and 126 Chinese lung cancer patients (case-control sets 1 and 2); 109 and 126 normal controls. PAH concentrations measured in plasma by BPDE Protein Adduct ELISA Kit.	blood<br><br>DNA Illumina Human Methylation450K and Illumina Human MethylationEPIC BeadChip<br><br>linear regression	NR	NA	Hypomethylation of *FAT1*
Ye, 2022[117]	1-OHP	293 workers (107 diesel exhausts and 186 coke oven). 203 unexposed controls. PAH measured in urine by high performance liquid chromatography.	blood<br><br>DNA methylation quantitative pyrosequencing in *TRIM36, RASSF1a & MGMT*<br><br>linear regression	NA	NA	Hypomethylation of *MGMT*
**PCBs congeners**						
Rusiecki, 2008[130]	28, 52, 99, 101, 105, 118, 128, 138, 153, 156,	70 Greenlandic Inuit, PCBs concentrations measured in plasma.	blood	PCB exposure associated with decreased	NA	NA

	170, 180, 183, 187		DNA methylation quantitative pyrosequencing in *Alu & LINE-1*<br><br>linear regression	global DNA methylation		
Kim, 2010[131]	74, 99, 105, 118, 126, 138, 153, 156, 157, 169, 170, 180, 189, 194, 206 & 209	86 healthy South Korean adults from a community-based survey. PCBs concentrations measured in plasma.	blood<br><br>DNA methylation quantitative pyrosequencing in *Alu & LINE-1*<br><br>linear regression	PCB exposure associated with decreased global DNA methylation	NA	NA
Itoh, 2014[129]	17, 28, 52/ 69, 48/47, 74, 66, 90/ 101, 99, 118, 114, 105, 146, 153, 164/163, 138, 128/ 162, 167, 156, 182/ 187, 183, 177, 180, 170, 189, 202, 198/ 199, 196, 203, 194, 208, 206,209	403 Japanese women who were controls in a breast cancer case–control study. PCBs measured in serum using high-resolution mass spectrometry.	blood<br><br>DNA Luminometric Methylation Assay (LUMA)<br><br>linear regression	PCB17, PCB52/69, PCB74, PCB114, and PCB183 associated with decreased global DNA methylation	NA	NA
Lind, 2013[132]	16 congeners<br>Dioxins- TCDD	1016 participants in the population-based Prospective Investigation of the Vasculature in Uppsala study. PCBs and dioxins measured in serum by high-resolution chromatography coupled to high-resolution mass spectrometry.	blood<br><br>DNA Luminometric Methylation Assay (LUMA)<br><br>linear regression	PCBs and dioxins exposure associated with increased global DNA methylation	NA	NA
Park, 2015[125]	15<br>12/15 congeners associated with MGMT methylation: 99, 105,118, 138, 153, 164, 172, 177, 178, 180, 183 & 187	368 South Korean participants without cancer. PCBs measured in serum by an isotope dilution method with gas chromatography-high resolution mass spectrometry.	blood<br><br>DNA methylation-specific PCR of *MGMT*<br><br>logistic regression	NA	NA	Hypermethylation of *MGMT*

Lee, 2017[126]	105, 118, 156, 157, 167	444 participants from the Korean Cancer Prevention Study. PCBs measured in serum by high-resolution mass spectrometry.	blood<br><br>DNA methylation quantitative pyrosequencing in *Alu & LINE-1*<br><br>linear regression	PCBs associated with decreased global DNA methylation in males; increased global DNA methylation in females	NA	NA
Georgiadis, 2019[127]	118, 138, 153, 156, 170 & 180	649 participants from the EPIC-ITALY & NSHDS prospective study cohorts. PCBs congeners assessed in blood plasma by gas chromatography.	blood<br><br>DNA Illumina Human Methylation 450K BeadChip<br><br>linear regression	NR	Hypermethylation of *REL*<br>Hypomethylation of *BCL11A, MITF*	Hypomethylation of *PTEN, ARHGEF12, FBXW7*
Curtis, 2021[128]	153, 138, 180, 118	641 participants from the Michigan PBB Registry. PCBs measured in serum using gas chromatography-tandem mass spectrometry.	blood<br><br>DNA Infinium Methylation EPIC BeadChip<br><br>linear regression	PCBs exposure associated with increased global methylation	From top 100 PCB-associated CpGs sites:<br><br>Hypermethylation of *LCK*<br><br>Hypomethylation of *ABL2, ARAF*	From top 100 PCB-associated CpGs sites:<br><br>Hypermethylation of *IGF2BP2*
**BENZENE**						
Bollati, 2007[95]	Benzene	155 Italian male workers (78 gasoline filling attendants and 77 urban traffic officers); 57 unexposed controls. Airborne benzene exposure measured passively during a work shift by thermal desorption followed by gas chromatography.	blood<br><br>DNA methylation-specific PCR of *p15 & MAGE-1*<br><br>linear regression	Benzene exposure associated with decreased global DNA methylation	Hypermethylation of *p15 (ABL1)*	NA
Xing, 2010[103]	Benzene	11 Chinese workers with benzene poisoning from a factory with documented benzene exposure for at least 6 months and clinical evidence; 8 healthy controls.	blood<br><br>DNA methylation-specific PCR of *p15 & p16*<br><br>Pearson correlation	NA	NA	Hypermethylation of p*16*^{INK4A} *(CDKN2A)*

Seow, 2012[102]	Benzene	158 Bulgarian workers (petrochemical); 50 unexposed controls. Benzene exposure measured in urine by high performance liquid chromatography and UV detection.	blood<br><br>DNA methylation pyrosequencing in *LINE-1& Alu;* methylation-specific PCR of *MAGE* & *p15*<br><br>ordinary least squares and beta-regression	Benzene exposure associated with decreased global DNA methylation	Hypermethylation in *p15 (ABL1)*	NA
Xing, 2013[104]	Benzene	77 workers (painters, shoe makers and printing makers), 41 of which with benzene poising; 25 unexposed controls. Benzene exposure assessed by factory/work unit/job title.	blood<br><br>DNA methylation-specific PCR of *BLM, CYP1A1, EPHX1, ERCC3, NQO1, NUDT1, p15, p16, RAD51, TP53, & WRAP53*<br><br>Pearson correlation	NA	NA	Hypermethylation of *ERCC3*
Yang, 2014[109]	Benzene	4 workers (paint sprayers) with benzene poisoning; 4 unexposed controls.	blood<br><br>DNA Illumina Human Methylation 450K BeadChip<br><br>Fisher's exact test, $\chi 2$ and hierarchical cluster	4297 hypermethylate d and 558 hypomethylated CpG sites among those with chronic benzene poisoning	Hypomethylation of *STAT3*	NA
Li, 2017[97]	Benzene	96 Chinese workers (petrochemical); 100 unexposed controls. Benzene measured in the air by solvent relief absorption gas chromatography; in urine by liquid chromatography/electrospray tandem mass spectrometry.	blood<br><br>DNA methylation-specific PCR of *MGMT* promoter<br><br>linear regression	NA	NA	Hypomethylation of *MGMT*
Zhang, 2017[105]	Benzene	410 Chinese workers (shoe manufacturers) with benzene poising; 102 unexposed controls. Benzene measured in the workplace air by gas	blood<br><br>MethylFlash Methylated DNA Quantification Kit	Benzene exposure associated with decreased	NA	NA

		chromatography with a flame ionization detector.	linear regression	global DNA methylation		
Zheng, 2017[107]	Benzene	41 Chinese workers (painting, shoe making and printing) with benzene poising; 35 unexposed controls.	blood<br><br>DNA methylation-specific PCR of *ERCC3*<br><br>linear regression	NA	NA	Hypermethylation of *ERCC3*
Jamebozorg, 2018[96]	Benzene	40 male workers (petrochemical); 31 unexposed controls. Benzene exposure by periodic environmental monitoring (not described in detail).	blood<br><br>DNA methylation-specific PCR of *p14 & p15*<br><br>Fisher's exact test	NA	Hypermethylation of *p15 (ABL1)*	NA
Ren, 2018[99]	Benzene	410 Chinese workers (shoe manufacturers); 102 unexposed controls. Benzene concentrations measured in air of workplaces by gas chromatography.	blood<br><br>MethylFlash methylated DNA quantification Kit<br><br>linear regression	Benzene exposure associated with decreased global DNA methylation	NA	NA
Rota, 2018[108]	Benzene	89 Italian male workers (petrochemical); 90 unexposed controls. Individual passive air sampler for exposure monitor; benzene measured by gas chromatography mass spectrometry.	blood<br><br>DNA methylation pyrosequencing in *LINE-1 & Alu* elements<br><br>linear regression	Benzene exposure associated with decreased global DNA methylation	NA	NA
Ren, 2019[100]	Benzene	141 Chinese workers (58 painting; 83 shoe factory); 48 unexposed controls. Benzene concentrations measured in air of workplaces by gas chromatography.	blood<br><br>DNA pyrosequencing in *LINE-1;* Methylation- specific PCR of *MGMT & hMLH1*<br><br>Fisher's exact test, $\chi 2$, hierarchical cluster analysis	Benzene exposure associated with decreased global DNA methylation	NA	Hypomethylation of *MGMT, MLH1(hMLH)*
Zhang, 2019[106]	Benzene	294 Chinese workers (shoe sewing, molding and packing); 102 unexposed controls. Occupational air benzene measured by gas	blood<br><br>MethylFlash Methylated DNA Quantification Kit	Benzene exposure associated with decreased	NA	NA

		chromatography with a flame ionization detector.	linear regression	global DNA methylation		
Ren, 2020[101]	Benzene	6 Chinese benzene-induced chronic poisoning patients; 6 controls. Benzene measured in the air at worksites.	blood<br><br>DNA Illumina HumanMethylation 450K BeadChip<br><br>linear regression	442/679 differentially methylated CpG sites were hypermethylated	Hypermethylation of *CSF3R, ALK, CSF1R, GNAS*<br><br>Hypomethylation of *NSD2*	Hypermethylation of *FAS, CDH1, FAT1*<br><br>Hypomethylation of *SMAD3, NRG1*
Philips, 2022[98]	Benzene	250 Chinese workers (shoe factory); 140 unexposed controls. More detailed exposure history from questionnaire.	blood<br><br>Illumina Human Methylation 450K BeadChip<br><br>linear regression	NR	Hypomethylation of *PRDM16*	NA

NA = not reported or assessed*; p16 = INK4A, CDKN2A; p53= TP53;* FFPE: Fixed, paraffin-embedded tumors
*per the NCG7.1 Network of Cancer Genes and Healthy Drivers
